# Plastic bronchitis: a narrative review of the classification methods and pathogenesis

**DOI:** 10.3389/fped.2026.1814321

**Published:** 2026-05-13

**Authors:** Haoyu Wang, Xiuhua Yu, Qianqian Lin, Fanyang Meng, Yanchun Li

**Affiliations:** 1 Department of Pediatric Respiratory Medicine, Children’s Medical Center, The First Hospital of Jilin University, Changchun, China; 2 Department of Radiology, The First Hospital of Jilin University, Changchun, China

**Keywords:** classification, eosinophilic, lymphatic, plastic bronchitis, review

## Abstract

Plastic bronchitis (PB) is a rare but potentially life-threatening disorder characterized by bronchial cast formation and airway obstruction. This narrative review synthesizes the evolution of PB classification and, in particular, integrates emerging evidence supporting a subtype-oriented framework—lymphatic vs. eosinophilic PB—based on distinct etiologies and pathophysiological mechanisms. We searched PubMed, Embase, Cochrane Library, and Web of Science for relevant clinical and experimental studies and reviews published up to February 2026, focusing on classification, mechanisms, diagnostic approaches, and therapies. We summarize key mechanistic pathways and the practical implications of advanced lymphatic imaging (e.g., DCMRL, CT lymphangiography) and immunopathology for improving subtype-specific diagnosis, guiding mechanism-based treatment beyond supportive care, and interpreting therapeutic responses. Finally, we highlight major knowledge gaps and propose priorities for future translational and clinical research to refine diagnostic criteria and develop precision therapies for PB subtypes.

## 1 Introduction

**Figure 1:**
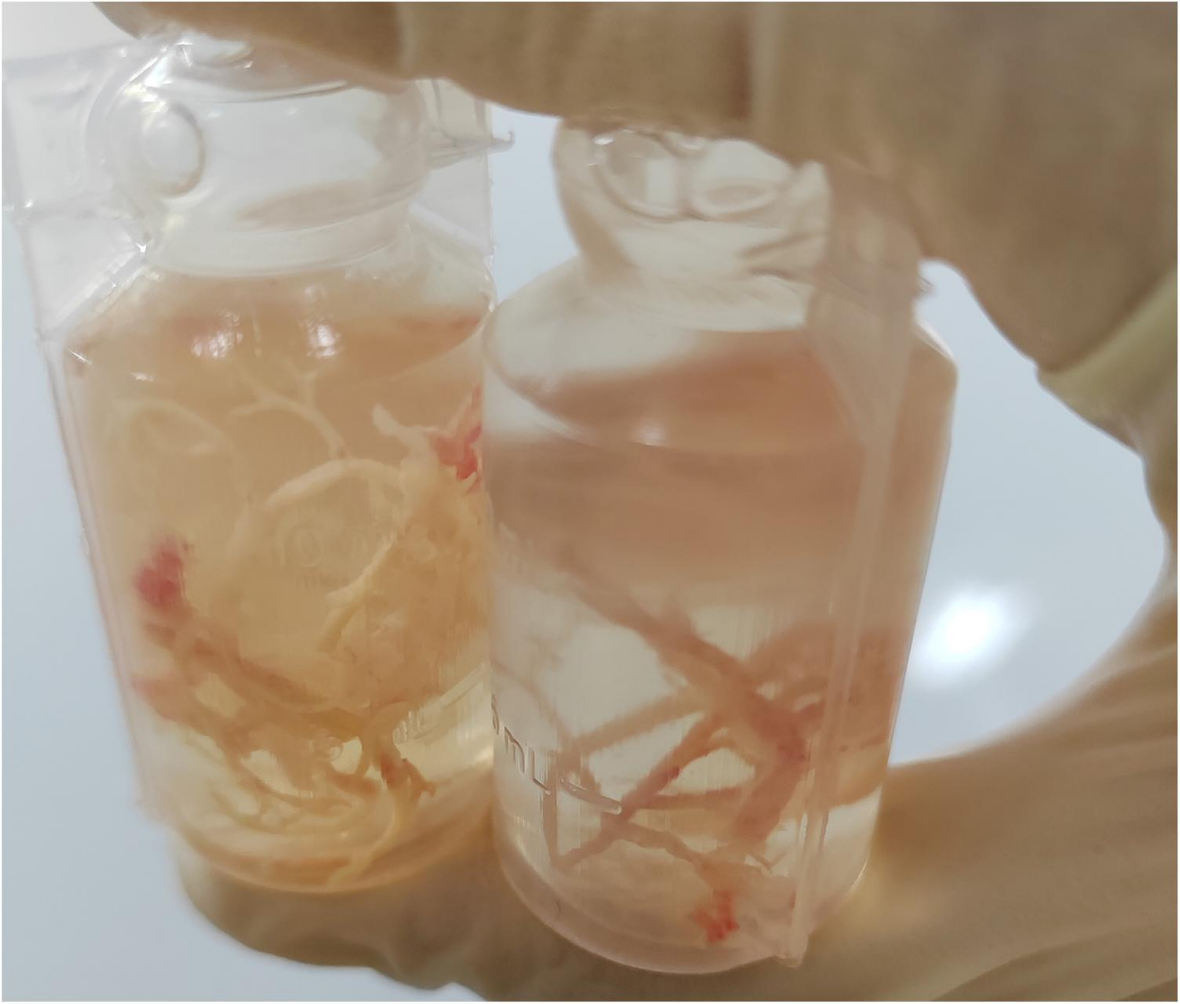
Representative gross appearance of a bronchial cast in plastic bronchitis. The cast shows a cohesive, branching structure that reproduces the architecture of the bronchial tree, a characteristic feature that helps distinguish true bronchial casts from ordinary mucus plugs.

Plastic bronchitis (PB) is a respiratory disorder characterized by the formation of large, cohesive bronchial casts arising from the solidification of airway secretions and/or inflammatory or lymphatic exudates. Unlike ordinary mucus plugging, which usually consists of softer and less organized secretions, PB involves firm or gelatinous casts that often reproduce the architecture of the tracheobronchial tree and may obstruct part or all of a bronchus (1, 2). To facilitate clinical recognition of this uncommon condition, a representative gross photograph of a bronchial cast is shown in Figure 1. The condition may therefore lead to partial or complete airway obstruction, causing ventilation dysfunction and, in severe cases, asphyxia (3).

PB has been reported in various clinical settings, though its overall incidence remains unclear. A single-center study in the United States found an incidence of 6.8 per 100,000 over 12 years, with prevalence rates in different patient cohorts: congenital heart disease (113 per 100,000), pneumonia and/or influenza (58 per 100,000), and asthma (29 per 100,000) (4). Only one death was reported among 14 patients, yielding a fatality rate of 7%. A multicenter study in Japan found a PB incidence of 0.1% (4/3,226) in post-Fontan patients, while an Australian study reported a rate of 1.8% (5, 6). In contrast, a multicenter study in China reported a 14.67% incidence in children with severe Mycoplasma pneumoniae pneumonia (SMPP), underscoring the clinical relevance of PB despite its rarity (7). A thorough understanding of the underlying mechanisms of PB is crucial for effective diagnosis and management; however, mechanistic studies remain limited and heterogeneous, highlighting the need for a mechanism-oriented framework to guide clinical decision-making. A conceptual flowchart of the literature selection process is provided in Supplementary Figure S1.

## 2 History of classification

In 1902, Beitmann (8) proposed the first classification scheme for plastic bronchitis (PB), based on its anatomical location and clinical presentation. In his review of 102 PB patients, he observed that the majority of cases were secondary, with asthma, tuberculosis, and organic heart disease identified as the primary causes. Later, in 1997, Seear et al. (9) introduced the most widely adopted classification method, dividing PB into two types: Type I (inflammatory) and Type II (acellular), based on the composition of casts. Type I casts are characterized by a high content of fibrin, low mucin levels, and the presence of Charcot-Leyden crystals, along with a dense eosinophilic inflammatory infiltrate. Type II casts, on the other hand, contain more mucin, less fibrin, and fewer cellular components. While this classification is straightforward, it has limitations. Notably, the inflammatory casts in Type I resemble those seen in asthma and allergic bronchopulmonary aspergillosis, and the chylous casts cannot be classified under this system (3, 10, 11). Additionally, the overlap between the two types complicates the differentiation process (3, 11). In 2005, Madsen et al. (10) proposed a new classification based on the presence or absence of structural heart disease. They further categorized PB with structural heart disease into mucinous casts, mucinous casts with inflammatory cells, and casts with both mucin and fibrin, while PB without structural heart disease was divided into lymphatic abnormalities/chylous casts, atopic disease/eosinophilic casts, and sickle cell acute chest syndrome with fibrinous casts, based on clinical and histological findings. However, this system excluded idiopathic cases and was not widely adopted in clinical practice. More recently, as research into the mechanisms of PB progressed, Dori and Itkin (12) identified PB in patients with lymphatic abnormalities as lymphatic plastic bronchitis (LPB). In 2023, Rubin (13) established a PB patient registry and specimen repository, which allowed for the collection of numerous cases. In these cases, PB patients without lymphatic abnormalities often exhibited eosinophils and their degradation products in their casts, leading Rubin to further categorize PB into LPB and eosinophilic plastic bronchitis (EoPB).

## 3 Lymphatic plastic bronchitis

The etiology of LPB is primarily associated with primary or secondary lymphatic vessel dysplasia. Primary lymphatic vessel dysplasia is most commonly observed in conditions such as diffuse pulmonary lymphangiomatosis, primary pulmonary lymphangiectasis, and certain lymphatic dysplasia syndromes, including Noonan syndrome (14, 15). Secondary lymphatic vessel dysplasia is primarily seen following congenital heart disease surgeries, such as the Fontan, Glenn, and Blalock-Taussig procedures (16). The exact mechanism by which infections lead to cast formation in LPB remains unclear. Some literature suggests that infections may contribute to LPB by triggering an inflammatory response, which in turn causes fibrin cross-linking in lymph fluid, promoting the formation of LPB casts (17). Additionally, cases of PB induced by silicosis have been reported, in which silicosis causes fibrosis in the thoracic lymph nodes, leading to significant lymph node involvement (18).

There is a paucity of literature on the pathological manifestations of LPB casts. Some reports indicate that lipids, fibrin, and mucoproteins are the primary components, with an increase in lymphocytes being a characteristic feature (18, 19). The thoracic duct (TD) is the largest lymphatic vessel in the human body, responsible for draining lymph from the left side of the chest into the venous system at the venous angle. The presence of valves in the lymphatic vessels prevents retrograde flow. Languepin et al. (19) observed lymphatic dilation in three children with PB and proposed that lymphatic fluid leakage into the bronchioles may contribute to the development of PB. Parikh et al. (20) ligated the TD in patients and improved their PB symptoms, providing evidence that abnormalities in the lymphatic system may cause PB. Imaging techniques play a crucial role in diagnosing abnormal pulmonary lymphatic flow. The advent of pedal lymphangiography (PL) allowed researchers to identify “lymphatic reflux”, a phenomenon where lymph fluid flows from the TD to the lung parenchyma (21, 22). In 2012, Nadolski et al. (23) advanced PL by introducing intranodal lymphangiography (IL) within the inguinal lymph nodes. However, the use of oil-based contrast agents, which had difficulty diffusing into smaller lymphatic vessels, limited further research into pulmonary lymphatic flow (24). Later, Dori et al. (25, 26) utilized dynamic contrast-enhanced MR lymphangiography (DCMRL) in a porcine model and applied it to children with congenital heart disease-related PB, revealing significant anatomical abnormalities in the lymphatic vessels of some PB patients. They performed IL on 18 PB patients and found that 16 had lymph flow from the TD to the peribronchial lymphatics and lung parenchyma. These abnormal lymphatic perfusions were classified into five types, and this phenomenon was termed “pulmonary lymphatic perfusion syndrome” (27). In 2016, Itkin et al. (12) demonstrated the presence of pulmonary lymphatic circulation disorders in adults and non-congenital heart disease-related PB patients, subsequently categorizing these patients as LPB. In 2022, O'Leary et al. (28) compared CT imaging and lymphoscintigraphy in 44 PB patients, showing significant differences in the CT images between LPB and non-LPB patients. This study supported the feasibility of LPB classification and proposed that one cause of PB and other lymphatic system-related diseases is the obstruction of the TD outlet or major branches, leading to lymphatic reflux from the TD to the mediastinum, lungs, pleura, and submucosa of the bronchi, resulting in corresponding pathological changes. This finding is consistent with the observations of Li et al. (29).

**Figure 2:**
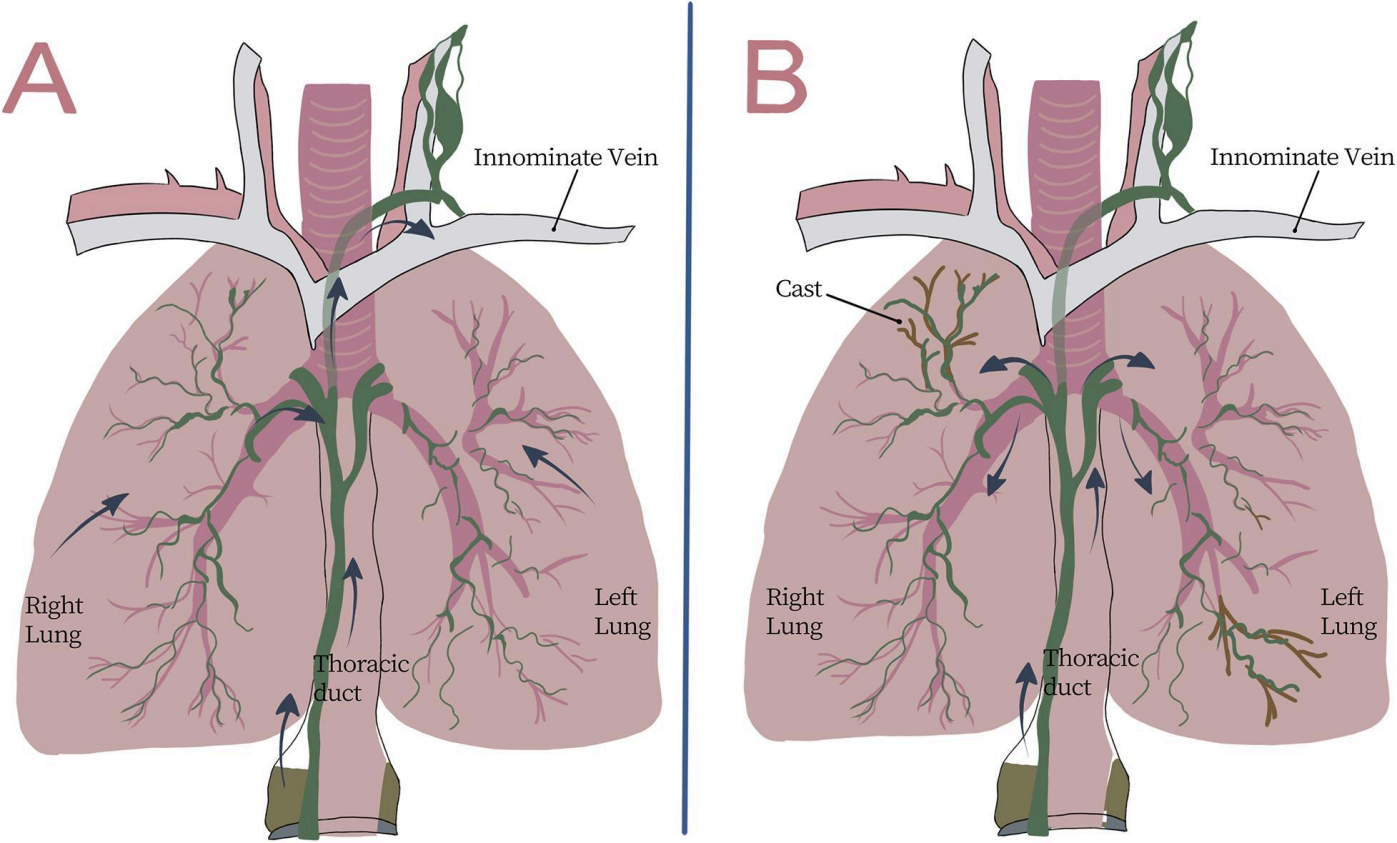
**(A)** Schematic diagram of normal pulmonary lymphatic flow. **(B)** Abnormal pulmonary lymph flow is characterized by the flow of pulmonary lymph from the thoracic duct to the pulmonary parenchyma.

Recent studies suggest that LPB can be considered a form of pulmonary lymphatic disorder (PLD), as advanced lymphatic imaging, such as DCMRL or CT lymphangiography, can demonstrate abnormal pulmonary lymphatic flow in this subtype, supporting the role of lymphatic reflux in cast formation (30, 31). Surgical interventions for congenital heart disease, primary lymphatic system disorders, and other lymphatic lesions can lead to elevated central venous pressure or obstruction of lymphatic vessels, which in turn increases pressure within TD. This results in lymphatic fluid reflux and the development of numerous small, dilated, and tortuous lymphatic channels extending into the peribronchial lymphatic vessels and lung parenchyma (Figure 2). Bronchial cast formation is thought to result from abnormal lymphatic perfusion within the bronchial submucosa, which leads to the gradual leakage of lymphocytes, proteins, and lipids into the bronchial lumen. These components subsequently dry, denature, and coagulate to form bronchial casts. These substances then dry, denature, and ultimately coagulate, forming bronchial casts. The identification of lymphatic system abnormalities as a cause of PB marks a significant advancement in the diagnosis and treatment of LPB. However, the precise mechanism through which lymphatic fluid refluxes into the bronchi and forms casts remains unclear. Future studies on LPB should focus on a more in-depth exploration of this mechanism and, based on the underlying etiologies of LPB, establish targeted treatment strategies. From a practical standpoint, LPB management typically proceeds from urgent airway clearance to etiologic phenotyping with advanced lymphatic imaging and, when feasible, targeted lymphatic interventions.

## 4 Eosinophilic plastic bronchitis

**Figure 3:**
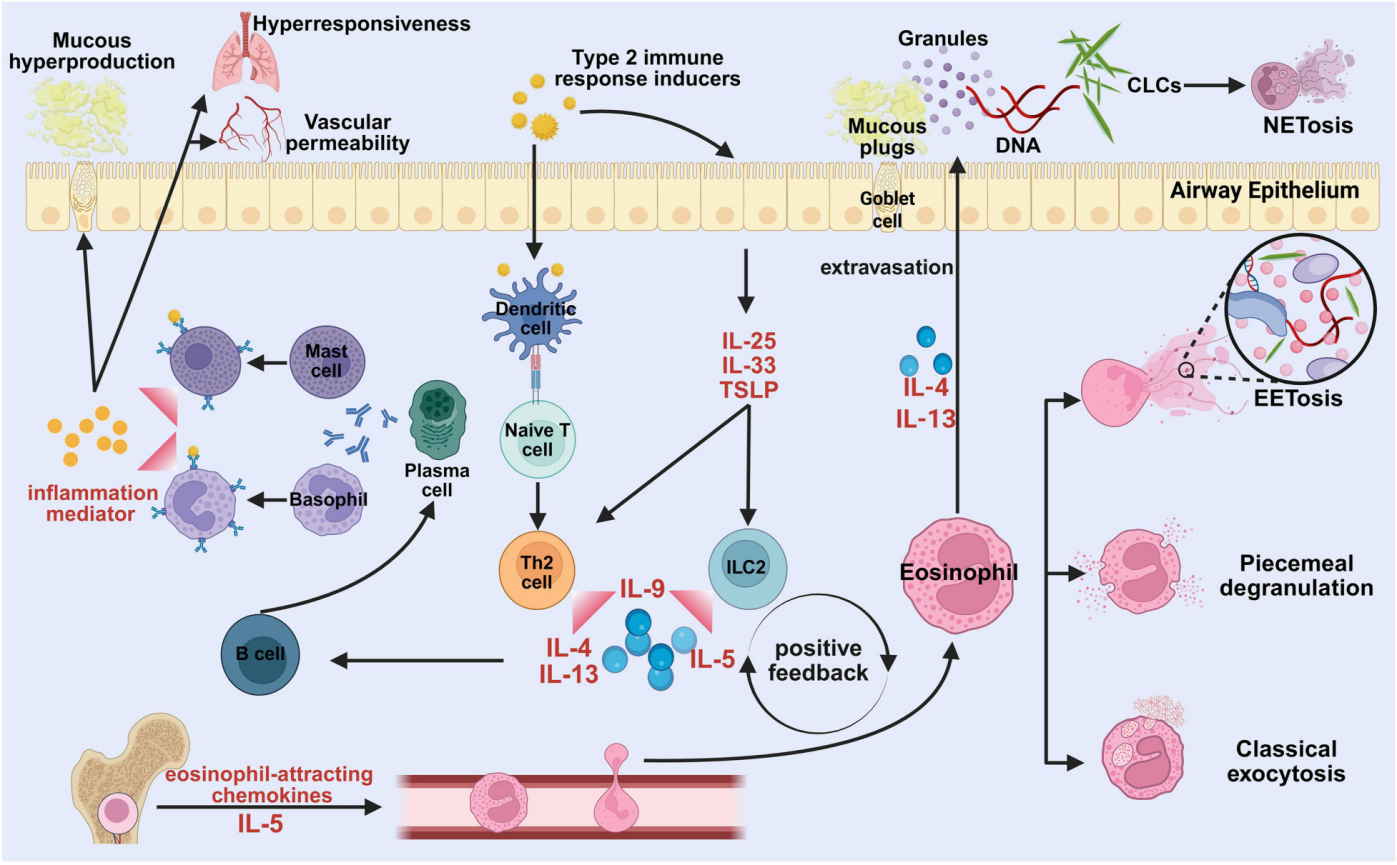
Proposed mechanism of cast formation under type 2 inflammation in eosinophilic plastic bronchitis. Considering the complexity of the stimuli that initiate type 2 immunity, we collectively refer to them as “type 2 immune response inducers”, which are typically categorized into macro-parasites and allergens. Kopp et al. (46) characterized their common feature as “tissue perturbation”. The intricate nature of type 2 immune activation may help explain the heterogeneity and complexity of primary diseases observed in PB patients. Nonetheless, the existence of a definitive causal relationship between these inducers and disease pathogenesis warrants further investigation.

In contrast to LPB, EoPB resembles the inflammatory (type I) casts described in the Seear classification. Asthma and atopic disease are common triggers, yet an increasing proportion of EoPB occurs in non-asthmatic patients with similar cast pathology (32–35). Infections are frequently reported as precipitants, but the causal pathway remains uncertain; in some cases, airway injury may instead be associated with neutrophil-predominant casts (36, 37). Histologically, EoPB casts are typically fibrin-rich and eosinophil-predominant and may contain Charcot-Leyden crystals and Curschmann spirals (38–42). Given the prominent fibrinous matrix, adjunctive antifibrin strategies proposed in the Denver classification and treatment guideline—such as airway-delivered alteplase to facilitate cast dissolution and inhaled heparin to reduce recurrent fibrin cast formation—may be considered when fibrin is a major cast component (43). Accordingly, when cast histology indicates a fibrin-predominant eosinophilic phenotype, acute bronchoscopic clearance remains central, while adjunct antifibrin strategies and type 2–directed anti-inflammatory therapy may be considered in selected patients. Evidence supporting eosinophil activation in cast formation includes the identification of eosinophil extracellular traps within post-influenza casts and endobronchial biopsies demonstrating eosinophilic inflammation (Figure 3) (44, 45).

Mechanistically, EoPB can be conceptualized as a type 2-biased inflammatory endotype in which epithelial injury by allergens or viruses triggers alarmins (IL-33, IL-25, and TSLP) via pattern-recognition signaling and activates the ILC2-dendritic cell-Th2 axis, promoting downstream type 2 cytokines (IL-4, IL-5, and IL-13) (46–48). IL-5 is central to eosinophil recruitment, maturation, activation, and survival in the airways (49, 50), and key regulatory receptors implicated in eosinophil biology include IL-5Ralpha, Siglec-8, and CRTH2 (51–53). IL-4 and IL-13 facilitate eosinophil extravasation and trafficking toward the airway epithelium (54), and eosinophils can secrete IL-5 in an autocrine manner that amplifies and sustains inflammation (50, 55). This framework is consistent with the frequent association of EoPB with asthma and other atopic disorders (56–58). Activated eosinophils degranulate and may undergo ETosis, releasing eosinophil extracellular traps together with cytotoxic mediators and galectin-10, which can crystallize into Charcot-Leyden crystals and further amplify type 2 inflammation (59–62). Compared with neutrophil extracellular traps, eosinophil traps exhibit higher viscosity (63, 64) and can provide a dense scaffold that traps fibrinous exudate and airway secretions, favoring cast solidification; CLCs may also promote neutrophil recruitment and NETosis, potentially contributing to mixed inflammatory patterns (65). Nevertheless, infection-associated neutrophil-predominant casts have been reported (37), indicating substantial heterogeneity and suggesting ETosis is not the sole determinant of cast formation. Under IL-5 and eosinophil-attracting chemokines, eosinophils traffic from the bone marrow into blood and rapidly migrate into tissues (66).

A complementary, but likely variable, contributor is airway secretory hyperresponsiveness and mucin dysregulation, which may increase cast burden particularly in mucinous/type II PB and some infection-associated cases (67, 68). Type 2 cytokines and allergic mediator release can drive goblet-cell hyperplasia and mucus hypersecretion (53, 69); the gel-forming mucins MUC5AC and MUC5B are key determinants of airway viscoelasticity (70), and increased expression has been reported in Mycoplasma pneumoniae pneumonia complicated by PB (71). In asthma, MUC5AC tends to predominate over MUC5B (72, 73), and experimental data suggest MUC5AC forms a stiffer and more viscoelastic gel (74). Charcot-Leyden crystals may promote goblet-cell metaplasia and upregulate MUC5AC expression (62), and may also structurally interact with mucus, increasing its density and impairing clearance (75). Importantly, lymphatic reflux does not appear to be a consistent feature of EoPB: in the largest recent series, MR lymphangiography performed in an EoPB patient was normal, and no abnormal lymphatic flow was identified (45). Overall, mechanistic links between these pathways and fibrin-rich cast formation require further clinical and experimental validation.

## 5 Discussion

Plastic bronchitis is a heterogeneous airway cast disorder in which a subtype-oriented framework can help translate mechanistic insights into practical decision-making. In lymphatic plastic bronchitis, advanced lymphatic imaging supports the concept that abnormal pulmonary lymphatic flow and lymphatic reflux contribute to cast formation, thereby providing a rationale for targeted lymphatic interventions in appropriately phenotyped patients. In eosinophilic plastic bronchitis, available histopathologic and mechanistic observations support a fibrin-rich, eosinophil-driven endotype in which extracellular traps and inflammatory exudates may provide a scaffold for cast solidification, highlighting the importance of cast composition in guiding adjunctive therapies and prevention strategies.

**Figure 4:**
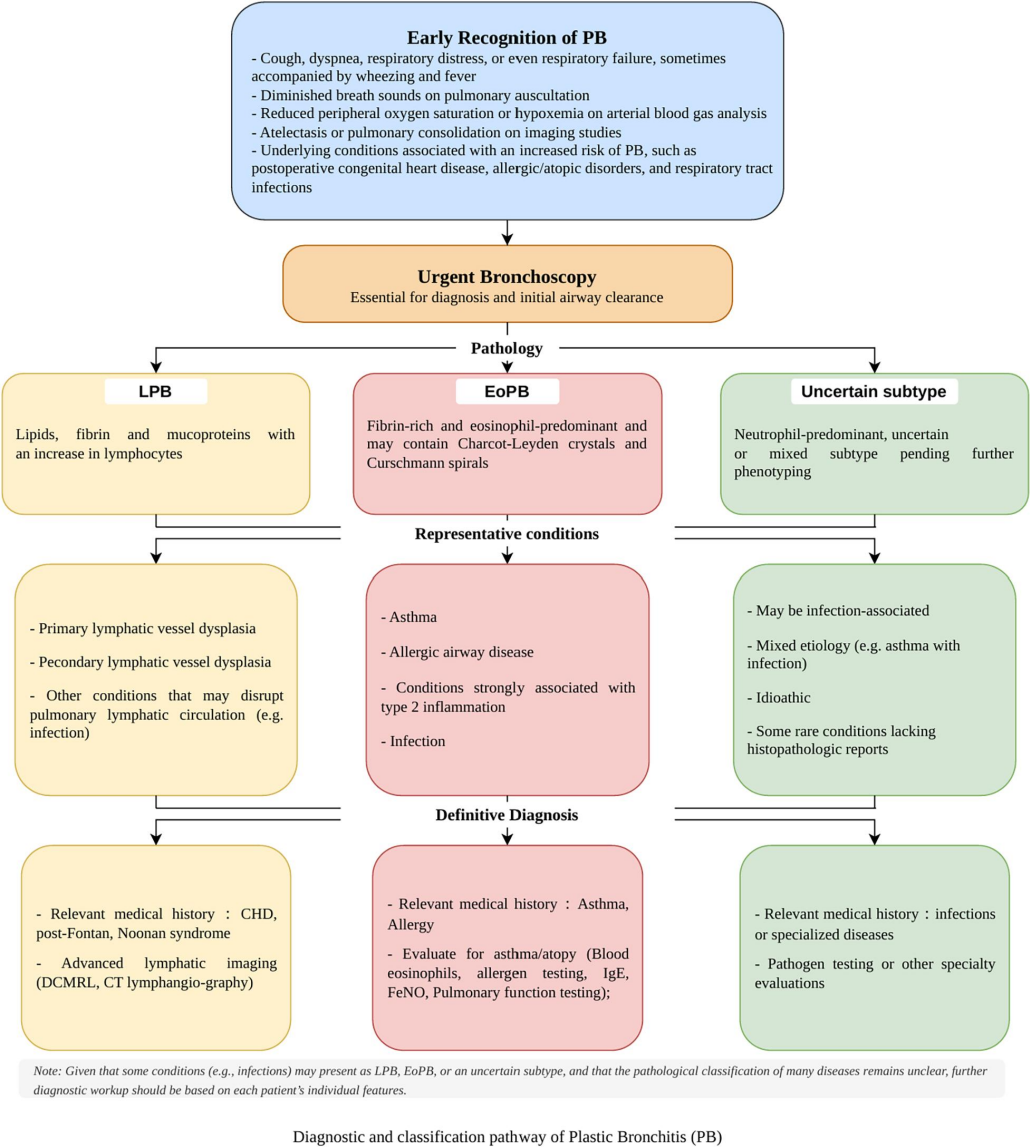
Diagnostic and classification pathway of plastic bronchitis (PB).

From a clinical perspective, the utility of this subtype-oriented framework becomes tangible when translated into a structured bedside diagnostic approach. In pediatric patients presenting with acute or recurrent airway obstruction, bronchoscopy assumes a central role both diagnostically and therapeutically (2, 76). It allows direct visualization of airway casts, confirming the presence, location, and morphology of bronchial obstruction, and simultaneously facilitates immediate removal to relieve respiratory compromise. Bronchoscopic sampling provides material for histopathologic evaluation, distinguishing lymphocyte- or eosinophil-predominant casts, and allows microbiologic analysis to identify potential infectious contributors. This procedure is crucial for differentiating true PB from severe mucus plugging, as PB casts typically exhibit cohesive, branching structures conforming to the bronchial architecture rather than amorphous mucus accumulation. Imaging studies, including chest radiography and computed tomography, serve as complementary tools to identify atelectasis, lobar collapse, or hyperinflation and to localize obstructed airways; advanced lymphatic imaging (e.g., DCMRL or CT lymphangiography) may be warranted subsequently when lymphatic PB is suspected. Moreover, Figure 4 provides a practical visual summary of the PB diagnostic and subtype-classification workflow, highlighting early clinical recognition, urgent bronchoscopy, and differentiation into lymphatic or eosinophilic PB to guide individualized treatment strategies. Accurate pathological classification of casts can help clinicians rapidly determine the underlying mechanism—whether inflammatory or lymphatic—thereby facilitating timely, individualized therapeutic strategies.

At the same time, the etiology of PB remains complex and multifactorial, and not all cases can be fully explained by the LPB/EoPB framework. In many patients, the precise process of cast formation remains incompletely understood, and the available literature suggests the existence of additional phenotypic patterns, such as neutrophil-predominant casts (77). Some patients with nephrotic syndrome may concurrently develop PB (78). It has been hypothesized that the pathogenesis in such cases may resemble that observed after the Fontan procedure, in that hypoproteinemia could promote fluid extravasation and secondary lymphatic abnormalities (78). Acute chest syndrome, a complication of sickle cell disease, may likewise precipitate PB through impaired mucociliary clearance, disrupted lymphatic circulation, and inflammatory responses; previous pathological examinations have demonstrated eosinophilic infiltration together with substantial fibrin deposition (79). In addition, tumors and other rare systemic disorders have also been implicated. Marll et al. (80), for example, reported PB in a patient with HIV-associated Kaposi's sarcoma. Collectively, these observations further support the view that PB should be regarded as a clinicopathologic syndrome with overlapping or incompletely defined mechanisms, and that, pending further phenotypic characterization, some patients may be more appropriately classified as having an uncertain or mixed subtype. To better illustrate this etiologic heterogeneity, Table 1 summarizes the major diseases and clinical settings associated with PB.

**Table 1:** Major etiologic categories, associated conditions, and plastic type in pediatric plastic bronchitis (“LPB/EoPB” indicates possible mechanisms and does not imply that both are always present; mechanism may vary depending on individual patient features).

Etiologic category	Representative conditions/clinical scenarios
Primary lymphatic vessel dysplasia	Diffuse pulmonary lymphangiomatosis, primary pulmonary lymphangiectasis, and certain lymphatic dysplasia syndromes, including Noonan syndrome
Secondary lymphatic vessel dysplasia	Following congenital heart disease surgeries, such as the Fontan, Glenn, and Blalock-Taussig procedures, trauma, lymphobronchial fistula
Atopic/eosinophilic airway disease–associated PB	Asthma (poorly controlled or acute exacerbation), atopic constitution, allergic airway disease, eosinophilic pneumonia
Infection-associated PB	Mycoplasma pneumoniae infection, viral infections (Influenza A virus, Influenza B virus, COVID-19, Human bocavirus, Respiratory syncytial virus, Parainfluenza virus, Adenovirus, Epstein–Barr virus, Rhinovirus, Human metapneumovirus, etc.), bacterial infections (Staphylococcus aureus, Streptococcus pneumoniae, Moraxella catarrhalis, Haemophilus influenzae, Acinetobacter baumannii, Pseudomonas aeruginosa, Streptococcus pyogenes, Bordetella pertussis, etc.), fungal infections (Aspergillus spp., Candida spp., etc.)
Sickle cell disease–associated PB	Acute chest syndrome
Pulmonary inhalation diseases	Silicosis, smoke inhalation
Pulmonary vascular diseases	Pulmonary vascular malformations, collateral vessels of main-pulmonary artery
Post pulmonary surgery	Lung transplantation, post-lobectomy, thoracotomy
Other rare or iatrogenic causes	HIV-associated Kaposi sarcoma, systemic lupus erythematosus, acute lymphoblastic leukemia, nephrotic syndrome
Idiopathic PB	No identifiable underlying disease

The current evidence base remains limited by the rarity of PB, variable terminology and phenotyping across reports, and the predominance of case reports and small series. Standardized reporting of cast histology, including fibrin vs. mucin predominance and detailed cellular composition, together with broader access to advanced lymphatic imaging and prospective registries, will be essential to validate subtype-defining mechanisms and to clarify which interventions are most effective for specific PB endotypes. Greater consistency in describing the diagnostic workup—particularly the timing of bronchoscopy, cast analysis, and subtype-oriented imaging—would also improve comparability across studies.

In summary, refining PB classification around reproducible subtype features—especially cast pathology, clinical context, and lymphatic imaging patterns—may improve diagnostic precision and support mechanism-based treatment. Future work should prioritize prospective multicenter registries, standardized histopathologic scoring of casts, clinically applicable diagnostic algorithms, and mechanistic studies, including animal models, to identify molecular drivers and develop targeted therapies for PB subtypes (81, 82).
