# Supplementary material for: Plastic bronchitis: a narrative review of the classification methods and pathogenesis
Source: Front Pediatr. 2026 May 13;14:1814321. doi: 10.3389/fped.2026.1814321 (PMC13212312; doi:10.3389/fped.2026.1814321)

**Supplementary Figure 1.** Conceptual literature selection process for this narrative review of plastic bronchitis.


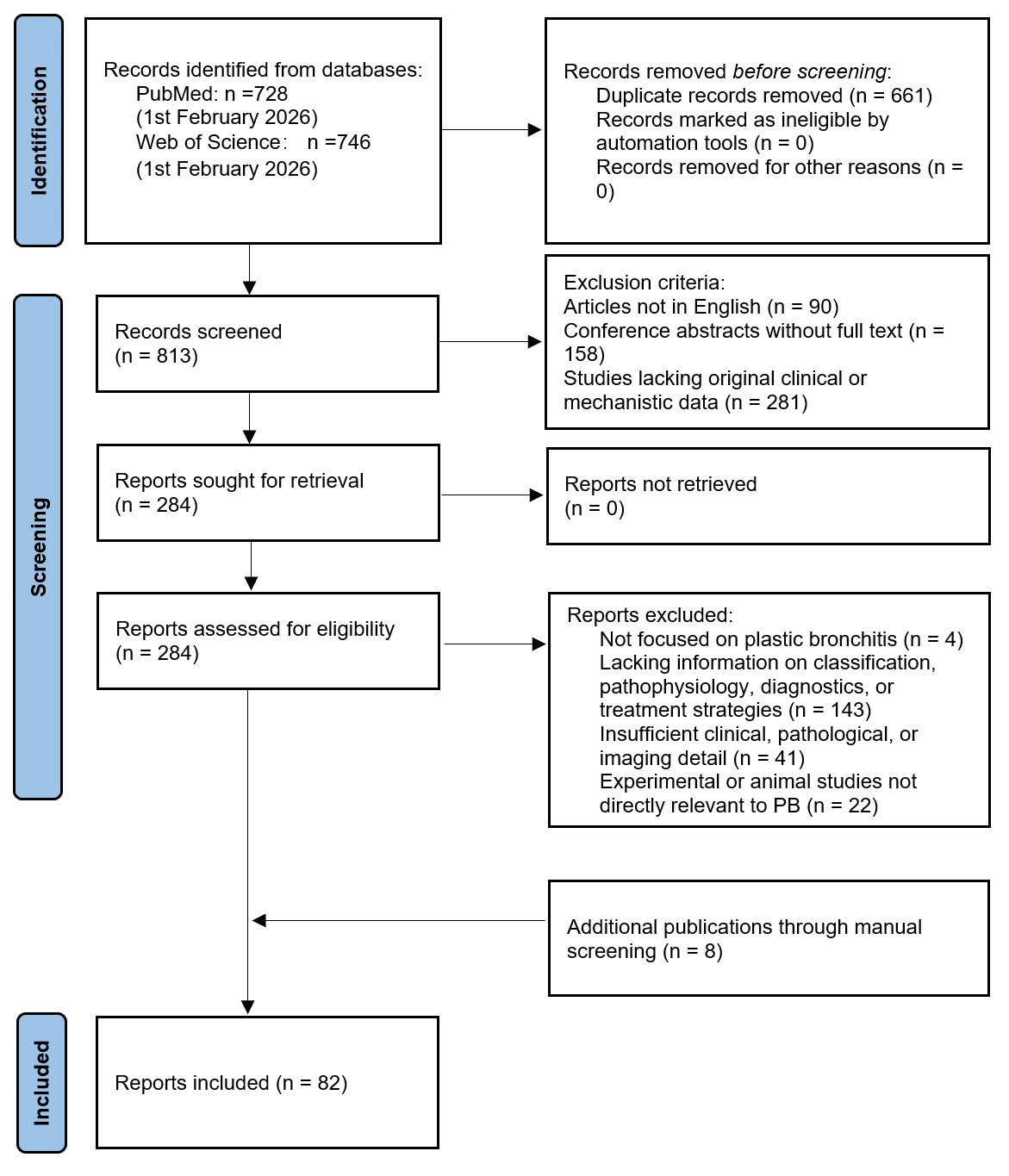

Supplement: Supplementary file 1 [file Supplementaryfile1.docx]
